# Partial tetrasomy of the proximal long arm of chromosome 15 in two patients: the significance of the gene dosage in terms of phenotype

**DOI:** 10.1186/s13039-015-0137-4

**Published:** 2015-06-25

**Authors:** Andras Szabo, Marta Czako, Kinga Hadzsiev, Balazs Duga, Katalin Komlosi, Bela Melegh

**Affiliations:** Department of Medical Genetics, University of Pecs, Szigeti 12, H-7624 Pecs, Hungary; Szentagothai Research Centre, Ifjusag 20, H-7624 Pecs, Hungary

**Keywords:** Array CGH, Epilepsy, Dysmorphism, 15q duplication syndrome, Supernumerary chromosome

## Abstract

**Background:**

Large amounts of low copy number repeats in the 15q11.2q13.3 chromosomal region increase the possibility of misalignments and unequal crossover during meiosis in this region, leading to deletions, duplications, triplications and supernumerary chromosomes. Most of the reported cases with epilepsy, autism and Prader-Willi/Angelman syndrome are in association with rearrangements of the proximal long arm of chromosome 15.

**Results:**

Here we report the first two unrelated Hungarian patients with the same epileptic and dysmorphic features, who were investigated by array comparative genomic hybridization (array CGH). By G-banded karyotype followed by FISH and array CGH we could detect partial tetrasomy of the 15q11.2q13.3 chromosomal region, supporting proximal 15q duplication syndrome. Findings of the array CGH gave fully explanation of the phenotypic features of these patients, including epileptic seizures, delayed development, hyperactivity and craniofacial dysmorphic signs. Besides the described features of isodicentric (15) (idic(15)) syndrome Patient 1. suffered from bigeminic extrasystoles and had postnatal growth retardation, which had been published only in a few articles.

**Conclusions:**

Dosage effect of some genes in the concerned genomic region is known, but several genes have no evidence to have dosage dependence. Our results expanded the previous literature data. We assume dosage dependence in the case of *CHRNA7* and *OTUD7A*, which might be involved in growth regulation. On the other hand increased dosage of the *KLF13* gene seems to have no direct causal relationship with heart morphology. The genomic environment of the affected genes may be responsible for the observed phenotype.

**Electronic supplementary material:**

The online version of this article (doi:10.1186/s13039-015-0137-4) contains supplementary material, which is available to authorized users.

## Background

Karyotyping and FISH are essential parts of the clinical evaluation of patients with epilepsy and dysmorphic features. FISH investigation is necessary in cases where (bi)satellited supernumerary chromosome is present in order to establish the chromosomal origin and the involvement of Prader-Willi/Angelman region (PWACR) in those with proven chromosome 15 (chr15) material. Increasing usage of array comparative genomic hybridization (array CGH) in the examination of patients with epilepsy and dysmorphic features allows the determination of the correlation between phenotype and genotype. Clinical studies investigated the function of chromosomal microarray analysis in the diagnosis of patients with epilepsy, and described an association between chromosomal copy number variations, intrachromosomal rearrangements and epileptic features [1]. Nevertheless, the genetic background of dysmorphic features remains unidentified despite the extensive investigation. This complex disease is associated with several previously described chromosomal alterations.

Small supernumerary marker chromosomes derived from the chr15 (sSMC(15)s) has been more frequently reported in connection with dysmorphic features and cause several phenotypes including intellectual disability, growth deficiency, triangular facies, and brachydactyly [2,3]. During the formation of this sSMC(15) a translocation occurs between the homolog chr15s, typically in the course of maternal meiosis, mediated by the low copy repeats being present within 15q11-q14. The following step is a non-disjunction and usually inactivation of one of the centromeres. Due to postzygotic correction a mosaic state is not uncommon. Within the 15q11q14 region five common breakpoints were identified (BP1 to BP5). The classification of sSMC(15)s is based on the euchromatin content (namely on the site of recombination). In contrast with the small sSMC(15)s containing the chromosomal material proximal to 15q12 and being clinically neutral, the larger sSMC(15)s resulting in partial trisomy or tetrasomy of Prader-Willi/Angelman region cause abnormal phenotype, in particular when of maternal origin [4]. Symptoms can vary depending on location of the breakpoints, copy numbers of the PWACR region and on the ratio of mosaicism. Clinical phenotype mostly includes early-onset central hypotonia, seizures, poor motor coordination, speech delay, moderate to severe learning disability, mild dysmorphic features, autism and schizophrenia [5–18]. Cytogenetically visible duplications (OMIM #608636) of this region are also reported, and lead to a disorder called 15q duplication syndrome, mostly manifested by neurobehavioral phenotypes [19]. These duplications occur mostly in two forms, including an extra isodicentric 15 (idic (15)) chromosome or an interstitial duplication 15. Based on previous literature data we can assume that patients with idic (15) of paternal origin seem to have normal development however, maternal aberrations can lead to developmental problems [4,20,21]. In most of the cases the 15q duplication syndrome is not inherited, but occurs randomly during the formation of reproductive cells. Wide range of developmental disabilities was experienced in the case of the individuals with 15q duplication syndrome. Examinations of the syndrome could not show an obvious correlation between the severity of the symptoms and the size of the duplicated region. Copy number changes of this chromosomal region from three to six have been reported in the literature [4,22,21]*.*

In order to establish the chromosomal content more precisely, we applied array CGH investigation of two Hungarian patients.

## Case Presentation

### Patients

We investigated 2 unrelated Hungarian patients with epilepsy and dysmorphic features.

**Patient 1.** is a 27 month old boy, first child of a non-consanguineous healthy young couple (father is 32 and mother is 31 years old), the mother has a boy from her first marriage. The family history and the pregnancy were unremarkable. He was delivered at the 40th week of gestation with a birth weight of 3200 g (25–50 pc) and an Apgar score 9/1 and 10/5, respectively. In the perinatal period cardiological examination was performed because of extrasystole. The Holter monitoring revealed bigeminic extrasystoles, but it regressed spontaneous shortly. His first epileptic seizure developed at 5.5 months of age, and he was never seizure free in spite of numerous different antiepileptic drug combinations. His seizure pattern is very variable, and at four years of age hyperactivity developed. His developmental milestones were delayed, he crept and stood at 18 months, but he could not walk. Speech development was delayed as well, only bubbling was present at 28 months.

At 28 months of age as he was first examined in our institution, his weight was 10 kg (<5 pc), height was 90 cm (50–75 pc) and OFC was 46 cm (<−2 SD). Flat occipital region, epicanthal folds, hyperteloristic eyes, crease of earlobes, broad nasal bridge, micrognathia and severe generalized hypotonia were present.

**Patient 2.** is a 17 months old girl from the second pregnancy (G2P1) of a non-consanguineous healthy young Caucasian couple (both parents are 32 years old), the family history is unremarkable. The girl was delivered at 41st weeks of gestation with a birth weight of 3520 g (75–90 pc) and Apgar score 1/10 and 5/10. The perinatal period was uneventful. Because of developmental delay she received neurohabilitation from the fourth month of age and West syndrome developed at the sixth month. Therefore vigabatrin treatment was administered, which caused significant seizure frequency reduction. Congenital brain malformation was supposed in the background but the performed brain MRI out of dilated frontal and temporal liquor spaces gave negative results. Her developmental milestones were delayed. At 12 months she sat alone and crawled, at 13 months she stood up, but at 17 months her walking capability was yet very unstable. Her speech development was severely delayed too.

She was referred to our institution because of epilepsy, dysmorphic features and developmental delay at the age of 17 months. At the examination her weight was 13 kg (90 pc), height 85 cm (75–90 pc), OFC 46 cm (−1 SD), and only mild craniofacial dysmorphic signs (prominent forehead, flat occiput, broad face, turned-up nose), broad thorax and small feet could be observed. In her neurological status severe generalized hypotonia, tiptoe walking and stereotype hand movements (clapping and wringing of the hands) were seen.

At her second examination at 29 month of age she could not walk alone and her walk was yet broad-base. Babbling started, but she had no clear words. Her stereotype movements were constant, but her seizure pattern and the frequency were changed. Therefore valproic acid was administered instead of vigabatrin.

## Results

### GTG banding and FISH

**Figure 1:**
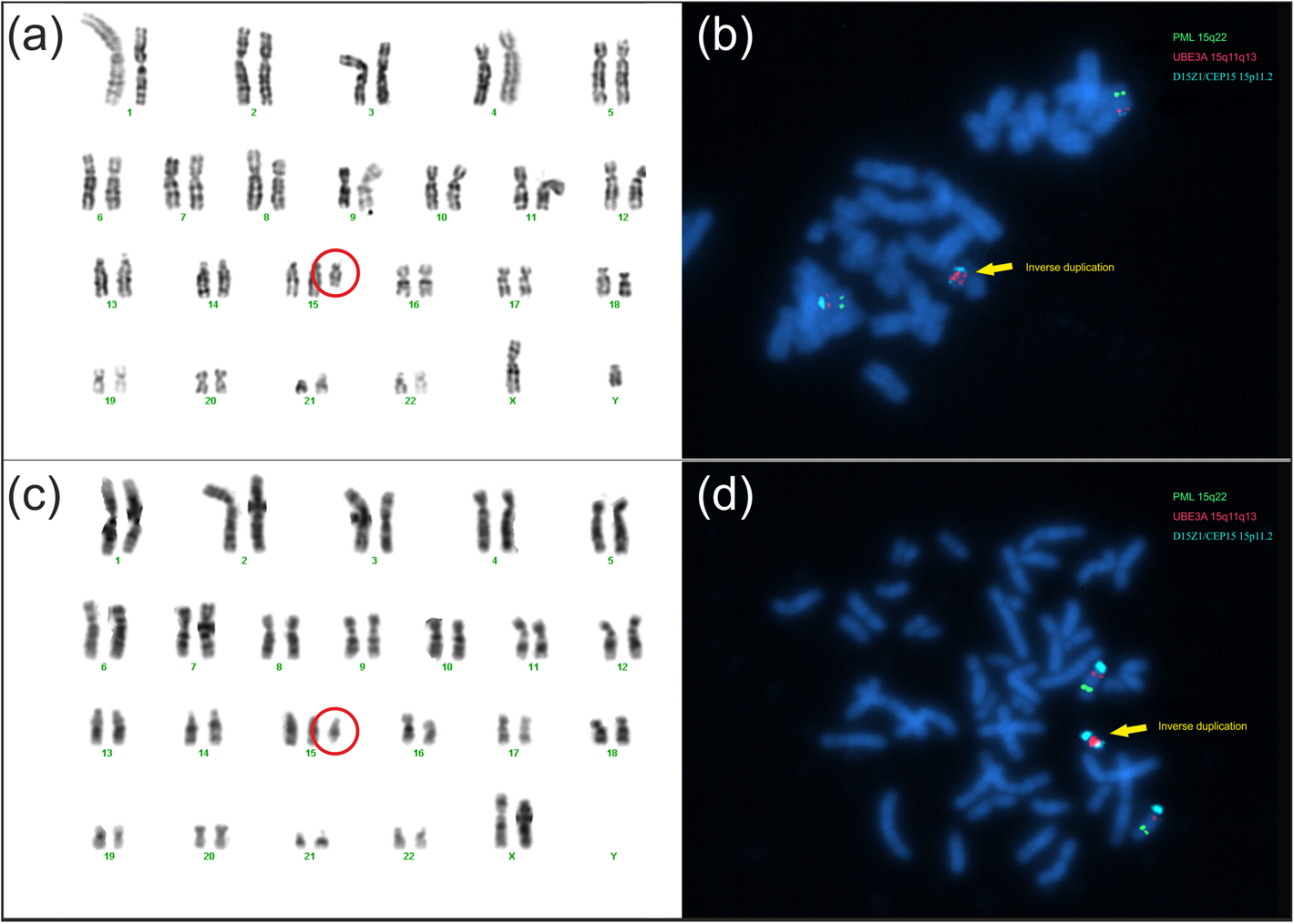
Karyotype and FISH analysis. **(a)** Karyotype of Patient 1. illustrates the supernumerary chromosome 15. **(b)** FISH analysis of Patient 1. by using probes for UBE3A locus (red) at 15q11q13, D15Z1/CEP15 locus (cyan) at 15p11.2 and PML locus (green) at 15q22 illustrates the tetrasomy of chromosome 15q11q13. **(c)** Karyotype of Patient 2. illustrates the supernumerary chromosome 15. **(d)** FISH analysis of Patient 2. by using probes for UBE3A locus (red) at 15q11q13, D15Z1/CEP15 locus (cyan) at 15p11.2 and PML locus (green) at 15q22 illustrates the tetrasomy of chromosome 15q11q13

GTG-banded chromosomes at the (550)-band level showed E-group sized supernumerary acrocentric marker chromosome in Patient 1. and an additional bisatellited chromosome in Patient 2. Because of the clinical symptoms/clinical indication metaphase FISH analyses of the UBE3A locus (Prader-Willi/Angelman Critical Region) was performed in both cases which showed the presence of both, the D15Z1 and UBE3A regions (each in two copies) on the supernumerary marker chromosomes in addition to the normal chromosomes 15 (four copies in all in each cells) (Figure 1). Thus, the karyotype of Patient 1. can be described as 47,XY,+psu idic (15) (pter → q14::q14 → pter) and that of Patient 2. is 47,XX,+idic (15)(pter → q14::q14 → pter). Both abnormalities resulted in tetrasomy of the 15q11q13 region.

Parental cytogenetic studies were normal.

### UPD study

Uniparental disomy of normal chromosomes 15 has been excluded using polymorphic STR markers. None of the two patients has uniparental disomy.

### Array-based copy number analysis

**Figure 2:**
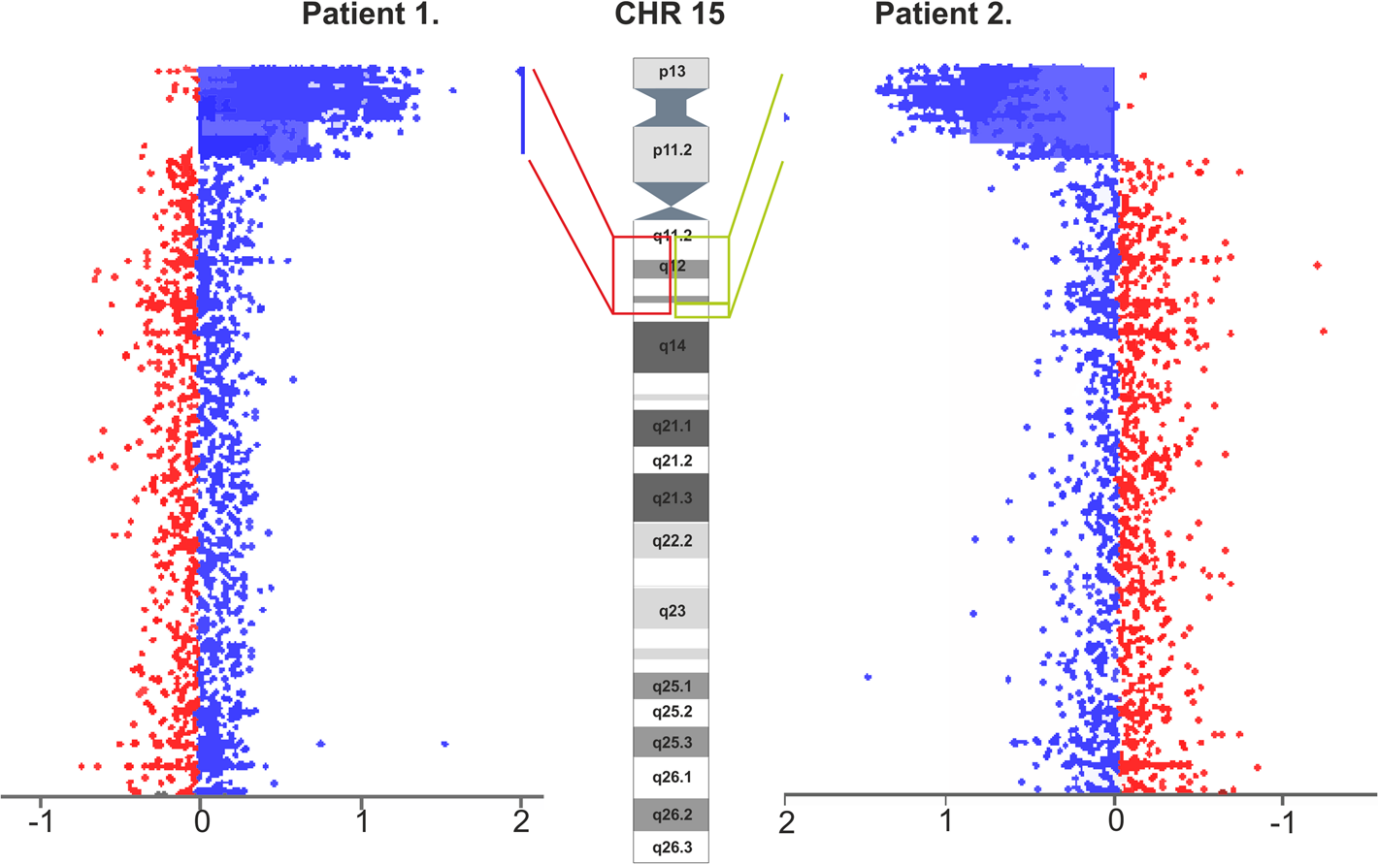
Array-CGH results of the two Hungarian cases. Figure shows the copy number gain of 15q11q13 region in Patient 1 (left) and in Patient 2 (right). Red box illustrates the localization of the copy number gain in **Patient1** on chromosome 15. Green box illustrates the positions of the copy number gains in **Patient 2**. on chromosome 15

Array CGH of Patient 1. showed an abnormal male array profile with copy number gain in the region **15q11.2q13.3 (22,765,628-32,445,252)**, while in Patient 2. an abnormal female profile with additional dosage of the regions **15q11.2q13.2 (22,765,628-31,183,907)** and **15q13.3 (31,261,835-32,861,626)**, respectively (Figure 2).

Both two investigated patients had gain in copy number of the 15q11.2q13.3 chromosomal region. Patient 1. had 4 copies of 15q11.2q13.3 (9.68 Mb, chr15:22,765,628-32,445,252), Patient 2. had 4 copies of 15q11.2q13.2 (8.42 Mb, chr15:22,765,628-31,183,907) and of 15q13.3 (1.6 Mb, chr15:31,261,835-32,861,626) chromosomal regions, demonstrating that the psu idic(15) contained two 9.68 Mb 15q11.2q13.3 segments (chr15:22,765,628-32,445,252) in Patient 1. and the + idic(15) contained two 15q11.2q13.2 segments with 8.42 Mb (chr15:22,765,628-31,183,907) and 1.6 Mb of 15q13.3 (chr15:31,261,835-32,861,626) regions in Patient 2.

## Discussion

Alterations of the 15q11.2q13.3 chromosomal region lead to a complex phenotypic variability, which greatly complicates the setting of a specific clinical diagnose. Here we present the results of the first study of array CGH analysis in Hungarian patients with epileptic seizures, delayed development, hyperactivity and craniofacial dysmorphic signs where the copy number gain of the region 15q11.2q13.3 (22,765,628-32,445,252) (Patient 1.), and that of 15q11.2q13.2 (22,765,628-31,183,907) and 15q13.3 (31,261,835-32,861,626) (Patient 2.) have been identified in the background of the abnormal phenotype.

**Figure 3:**
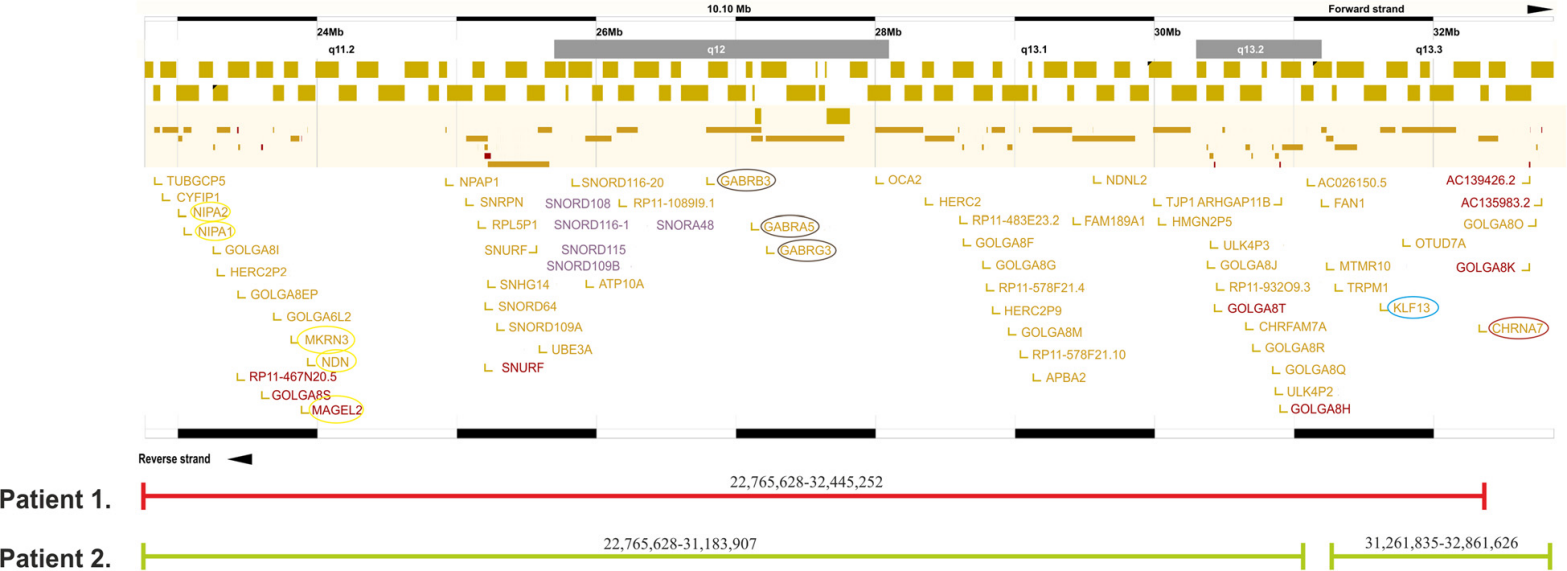
Ensembl results of duplicated regions and the concerned genes. (http://grch37.ensembl.org/Homo_sapiens/Location/Overview?r=15%3A22765628-32861626) Red line shows the duplicated 15q11q13 region of **Patient 1**. Green lines show the duplicated regions of **Patient 2**. The upper part of the figure illustrates the affected genes. (Red: protein coding; Yellow: merged Ensembl and Havana genes; Purple: RNA genes) The yellow circles indicate the genes might be involved in growth regulation. The brown circles indicate the genes might be involved in GABA mediated antiepileptic drug therapy. The blue circle indicates the KLF13 gene, which might be associated to cardiac malformations. The claret circle indicates the CHRNA7 gene, which might be associated with neuropsychiatric phenotypes

All of the protein coding genes, occurring in the 15q11.2q13.3 (22,765,628-32,861,626) duplicated region are shown in the Additional file 1: Table 1 and Figure 3*.* Genes within the affected regions are significantly linked to identified clinical disorders, mainly developmental, hereditary and neurological disorders. (Additional file 2: Table 2).

Based on our findings and previous literature data, we concluded, that most of the genes within the affected regions are significantly associated with the phenotypic features of the two analyzed patients. Hypotonia, delayed motor milestones (sitting up, walking), delayed language development, progressed hypertonia, and similar facial characteristics, including flat nasal bridge, epicanthic and hyperteloristic eyes in Patient 1. can assume a 15q duplication syndrome that affected the 15q11.2q13.3 (22,765,628-32,445,252) chromosomal region [21]. In addition to the symptoms were seen in Patient 1., Patient 2. represented typical Angelman like features including small feet, turned-up nose, tiptoe walking and stereotype hand movements (clapping and wringing of the hands).

After uneventful prenatal period, both of our patients had normal birth weight. By comparing our cases with the recently published patients investigated by array CGH and affected by 15q duplication syndrome we could identify more defined postnatal growth retardation in the case of Patient 1. (Additional file 3: Table 3). At 28 months of age his weight was only 10 kg (<5 pc), height was 90 cm (50–75 pc) and OFC was 46 cm (<−2 SD) comparing to Patient 2. with a 13 kg (90 pc) weight at age 17 months. On the contrary of the previous reported cases our Patient 1. had normal birth weight and the growth delay occurred subsequently. Proportional short stature was mentioned previously in many cases, but postnatal growth delay associated with low weight was observed to the best of our knowledge only in one 15q13.3 duplicated patient (DECIPHER), in which *CHRNA7* and *OTUD7A* genes have found within the duplicated chromosomal region. The role of the cholinergic nicotinic receptor *CHRNA7* gene has been attributed in neuropsychiatric disorders, epilepsy, cognitive impairment, furthermore, it is essential for inhibiting cytokine synthesis by the cholinergic anti-inflammatory pathway [23]*.* Dosage sensitivity of the gene has been reported in patients with 15q13.3 deletion in several cases, among them in a West-syndrome patient recently [24], and proved by Valbonesi et al. [25] in a cohort of ADHD patients where deletion and the resulting lower gene expression level was investigated. In a three-generation family with various neuropsychiatric phenotypes, copy number gain of *CHRNA7* showed co-segregation with the symptoms [26]*.* Our data/cases provide further evidence for the pathogenicity of copy number gain. Literature data of the intronless *NDN, MAGEL2, MKRN3* and the *NIPA1, NIPA2* genes confirm that these genes are involved in growth regulation, nervous system development or transcription. Deletion of the genes in mice and humans presents failure in nervous system development and growth regulation [27–29]*.* Increased dosage of the mentioned genes could play a role in the development of postnatal growth retardation.

The group of congenital heart defects is one of the most common causes of infant morbidity and mortality with an estimated prevalence of 1-5% of life births. Congenital heart defects caused by idic (15) duplication syndrome occur in about 25% of the cases [4] and 7-18% of patients with the 15q13.3 deletion syndrome (OMIM 612001) had heart defects [30,17,31,32]*.* Clinical findings from 15q microdeletion cases pointed that mitral valve prolapse, slightly enlarged left ventricle, tetralogy of Fallot and right cardiac hypoplasia with severe tricuspid stenosis could develop by the deletion of the *KLF13* gene of the BP4BP5 chromosomal region. *Kruppel-like transcription Factor 13* is a well conserved gene, which encodes a member of the Kruppel-like family of zinc-finger proteins. The protein is identified as a regulator of cardiac gene expression and heart morphogenesis [33,17]*.* Genetic studies in the Xenopus embryos also demonstrated a requirement for *KLF13* in cardiac progenitor cell proliferation and heart morphogenesis [34]*.* In *KLF13* functional knockout Xenopus embryos normal initial heart development was observed but, subsequently, the heart was visibly smaller and showed several defects including a lack of ventricular trabeculation, atrial septal defects, delayed atrioventricular cushion formation and maturation of valves [34]. Derwińska et al. presented a 1.6 Mb 15q13.3 duplication in a patient having cardiac phenotype and suggested that increased dosage of *KLF13* could be the causative of cardiac anomalies [35]*.* In our case neither patient showed cardiac anomalies, although Patient 1. did present bigeminic extrasystole. Based on the limited data available and on our results, we cannot support Derwińska’s findings about the association between the increased *KLF13* dosage and cardiac malformations.

Concerning the dosage sensitivity Hamid et. al. reported in the context of pericentric human chromosomal regions, that dosage dependent and dosage independent genes could stand in the background of the clinical effects of the sSMCs. He stated, that sSMCs containing only dosage independent genes could be harmless, while dosage dependent genes in the marker chromosomes could lead to clinical problems [36].

Regarding to the epilepsy specific treatment of the patients, antiepileptic drug therapy, using gamma aminobutyric acid transaminase inhibitor vigabatrin and valproic acid was effective. GABA acts at inhibitory synapses in the brain by binding to specific transmembrane receptors. Both of our patients have a gain in the GABA receptor subunit coding GABRB3, GABRA5 and GABRG3 genes, which could lead to an increased GABA receptor level. The used antiepileptic drugs increase the level of the GABA protein, by inhibiting the GABA metabolism of GABA transaminases. The higher receptor number associated with the increased GABA level could be a possible explanation for the reduced seizure frequency due to the successful treatment [37–39]*.*

An other database was available [40] containing data of 43 cases with inv dup (15) and seizures. However in this database 42 cases were investigated only with simple karyotyping and only one with array CGH. Additional file 3: Table 3. contains this case. The other 42 patients, thus could be compared to our cases only based on the phenotype. Phenotypic manifestation was mostly the same in our cases and the examined patients, except the presence of defined postnatal growth retardation in the case of Patient 1. The lack of array CGH results could not allow the clarification of the gene-phenotype relationship.

## Conclusions

We identified the first two array CGH examined Hungarian patients with tetrasomy of the proximal region of chromosome 15. Alterations of the 15q11.2q13.3 chromosomal region cause complex phenotypic variability, which greatly complicates the setting of a specific clinical diagnose. FISH analysis followed by array CGH provided clinically relevant information of the investigated patients. The copy number gain of the 15q11.2q13.3 chromosomal region could explain the phenotypic presentations characteristic for idic (15) syndrome observed in the patients, including epileptic seizures, delayed development, hyperactivity and craniofacial dysmorphic signs.

In case of a number of genes in the affected chromosomal regions dosage effect evidence was reported, however for certain genes, dosage dependence is not proven. Based on our findings, we could provide additional information for previous literature data in case, that increased dosage of *CHRNA7* and *OTUD7A* genes could lead to postnatal growth delay, so dose dependence of these genes could be assumed. In contrast, dosage dependence of the *KLF13* gene in a patient with an 1.6 Mb duplication involving the *KLF13* gene and in *KLF13* knockout Xenopus embryos was presumable, while none of our patients with higher copy number of *KLF13* showed cardiac anomalies. Thus, our results could not verify the dosage dependence of the *KLF13* gene. However it could be concluded, that not only the size of the duplicated region, but the gene content could be responsible for the whole clinical manifestation. Based on our results we can assume, that in case of these genes the development of the expected clinical features could be much better influenced by the copy number of the genes and their genomic environment, than only the size of the duplicated genetic material itself. Nevertheless, in the case of *NDN, MAGEL2, MKRN3* and the *NIPA1, NIPA2* genes confirmation is needed for the causal relationship between copy number gain and postnatal growth delay.

Further investigations are necessary to get better knowledge regarding to the association between the detailed genes and the phenotypes they might cause and to the dosage sensitivity of individual genes affected by the aberrations.

## Materials And Methods

### GTG banding

Karyotyping of the patient was performed by Giemsa–Trypsin (GTG) banding from peripheral blood lymphocytes using standard procedures [41].

### Fluorescent in situ hybridization (FISH)

We performed fluorescence in situ hybridization for the investigation of the supernumerary marker chromosome, using the 15q11-q13 (D15S10/UBE3A) Prader-Willi/Angelman probe with 15p11.2 (D15Z1/CEP15) and 15q22 (PML) as control probes (Vysis, Abbott Laboratories, Abbott Park, Illinois, U.S.A.) [42].

### Uniparental disomy study

Uniparental disomy of chromosomes 15 was examined using previously reported D15S11, D15S97, D15S122, D15S113, D15S659, D15S128, D15S210, D15S165, D15S10 and GABRB3 polymorphic STR markers [43,44].

### Array CGH

Agilent Human Genome Unrestricted G3 ISCA v2 Sureprint 8x60K oligo-array (Amadid 031746) (Agilent, Santa Clara, CA) was used for array CGH [45]. This high resolution microarray contains 18,851 60-mer oligo probes in ISCA regions + 40,208 backbone probes including coding and non-coding genomic sequences with an average 60 KB overall median probe spacing, respectively (higher in ISCA regions).

DNA purification from blood was performed using the NucleoSpin®Dx Blood DNA Purification Kit (ThermoFisher Scientific, Waltham, MA) applying the protocol of the producer. We measured the concentration and purity of the isolated DNA with the NanoDrop spectrophotometer. For labelling and for hybridization we used the Agilent Oligonucleotide Array-Based CGH for Genomic DNA Analysis – Enzymatic Labelling Protocol. 1 μg from the patient DNA and from a sex-matched reference DNA were digested for 2.5 hrs at 37 °C with AluI and RsaI enzymes. For the labelling of the digested DNA we applied random priming using the Agilent Genomic DNA Labelling Kit (Agilent, Santa Clara, CA). Patient and control DNA were labelled differently. Cy5-dUTP was used for labelling the patient samples and Cy3-dUTP for the control samples. Labelled DNA samples were purified by Amicon Ultra AU-30 filters. The patient and sex-matched reference samples were combined and cohybribized at 65 °C for 24 hrs rotation with 50 μg Human Cot-1 DNA. Washing step was performed using the Agilent Protocol v7.2. Array image was obtained using an Agilent laser scanner G2565CA and analysed with the Agilent FeatureExtraction software (v10.10.1.1.).

The results were presented by Agilent Cytogenomics software (v2.5.8.11). DNA sequence information refers to the public UCSC database. The detected chromosomal changes were aligned to known aberrations listed in publicly available databases. We used the DECIPHER (Database of Chromosomal Imbalance and Phenotype in Humans using Ensembl Resources), the Database of Genomic Variants, Ensembl and ECARUCA.

## Consent

Written informed consent was obtained from the patient for publication of this Case report and any accompanying images. A copy of the written consent is available for review by the Editor-in-Chief of this journal.

## Additional files

Additional file 1:
**Table 1.** Function of protein coding genes of the 15q11.2q13.3 (22,765,628-32,861,626) chromosomal region according to Decipher. (https://decipher.sanger.ac.uk/) Notes: Bold: OMIM genes; Italic: Morbid genes; HI(D): Haploinsufficiency scores from Decipher: values from 0-100%. Low values (0-10%) indicate more likely to exhibit Haploinsufficiency. The **α** labeling indicates the genes might be involved in growth regulation. The **β** labeling indicates the genes might be involved in GABA mediated antiepileptic drug therapy. The **γ** labeling indicates the KLF13 gene, which might be associated to cardiac malformations. The **δ** labeling indicates the CHRNA7 gene, which might be associated with neuropsychiatric phenotypes. Additional file 2:
**Table 2.** The list of the genes located in the duplicated regions and the associated diseases. Additional file 3:
**Table 3.** Comparing clinical features of array CGH investigated 15q11q13 duplication patients and our cases. α labeling indicates the postnatal growth retardation phenotype, while **γ** labeling indicates the cardiac symptom of Patient 1.

## Abbreviations

Array CGH: Array comparative genomic hybridization
idic(15): Isodicentric chromosome 15
PWACR: Prader-Willi/Angelman critical region
Chr15: Chromosome 15
sSMC(15): Small supernumerary marker chromosome derived from chromosome 15
FISH: Fluorescent in situ hybridization
ASD: Atrial septum defect
VSD: Ventral septum disease
MRI: Magnetic Resonance Image
EEG: Electroencephalography
GTG banding: Giemsa Banding
GRCh37:hg19: Genome Reference Consortium human 37/human genome 19
UCSC database: University of California, Santa Cruz Genome Browser database
DECIPHER: Database of Chromosomal Imbalance and Phenotype in Humans using Ensembl Resources
DGV: Database of Genomic Variants
ECARUCA: European Cytogeneticists Association Register of Unbalanced Chromosome Aberrations
